# Supplementary material for: Medicarpin and Homopterocarpin Isolated from Canavalia lineata as Potent and Competitive Reversible Inhibitors of Human Monoamine Oxidase-B
Source: Molecules. 2022 Dec 28;28(1):258. doi: 10.3390/molecules28010258 (PMC9822396; doi:10.3390/molecules28010258)
Supplement: Supplementary file 1 [file molecules-28-00258-s001.zip › molecules-2071925-supplementary.pdf]

molecules

SUPPORTING INFORMATION

Medicarpin and Homopterocarpin Isolated from *Canavalia lineata* as Potent and Competitive Reversible Inhibitors of Human Monoamine Oxidase-B

Jong Min Oh [1], Hyun-Jae Jang [2], Myung-Gyun Kang [3], Seul-Ki Mun [1], Daeui Park [3], Su-Jin Hong [2], Min Ha Kim [4], Soo-Young Kim [4], Sung-Tae Yee [1], Hoon Kim [1,*]

[1] Department of Pharmacy, and Research Institute of Life Pharmaceutical Sciences, Sunchon National University, Suncheon 57922, Republic of Korea
[2] Natural Product Research Center, Korea Research Institute of Bioscience and Biotechnology, Cheongju, 28116, Republic of Korea
[3] Department of Predictive Toxicology, Korea Institute of Toxicology, Daejeon 34114, Republic of Korea
[4] National Institute of Biological Resources, Environmental Research Complex, Incheon 22689, Republic of Korea
* Correspondence: hoon@scnu.ac.kr (H.K.); Tel.: +82-61-750-3751

Table of Contents

Figure S1. Separation scheme of compounds (**PN1–PN13**) from *Canavalia lineata* pods. 2

Figure S2. MS and UV chromatogram of *C. lineata* pod extract using UPLC-QTOF-MS. 3

Figure S3. UPLC-QTOF-MS spectrum of rutin (**1**). 4

Figure S4. UPLC-QTOF-MS spectrum of (2*R*,3*R*)-3-hydroxy 7-*O*-*β*-D-glucopyranoside-6-methoxy-flavanone (**2**). . 5

Figure S5. UPLC-QTOF-MS spectrum of (–)-syringaresinol-4-*O*-*β*-D-glucopyranoside (**3**). 6

Figure S6. UPLC-QTOF-MS spectrum of ononin (**4**). 7

Figure S7. UPLC-QTOF-MS spectrum of syringaresinol (**5**). 8

Figure S8. UPLC-QTOF-MS spectrum of (2*R*,3*R*)-3,7-dihydroxy-6-methoxyflavanone (**6**). 9

Figure S9. UPLC-QTOF-MS spectrum of cajanin (**7**). 10

Figure S10. UPLC-QTOF-MS spectrum of medicarpin (**8**). 11

Figure S11. UPLC-QTOF-MS spectrum of prunetin (**9**). 12

Figure S12. UPLC-QTOF-MS spectrum of 7,4'-dimethyl-3'-hydroxygenistein (**10**). 13

Figure S13. UPLC-QTOF-MS spectrum of 7,4'-dimethoxyisoflavone (**11**). 14

Figure S14. UPLC-QTOF-MS spectrum of pterocarpin (**12**). 15

Figure S15. UPLC-QTOF-MS spectrum of homopterocarpin (**13**). 16

Figure S16. IC_{50} curve of medicarpin (**8**) for hMAO-B. 17

Figure S17. IC_{50} curves of homopterocarpin (**13**) for hMAO-A (**A**) and hMAO-B (**B**). 18

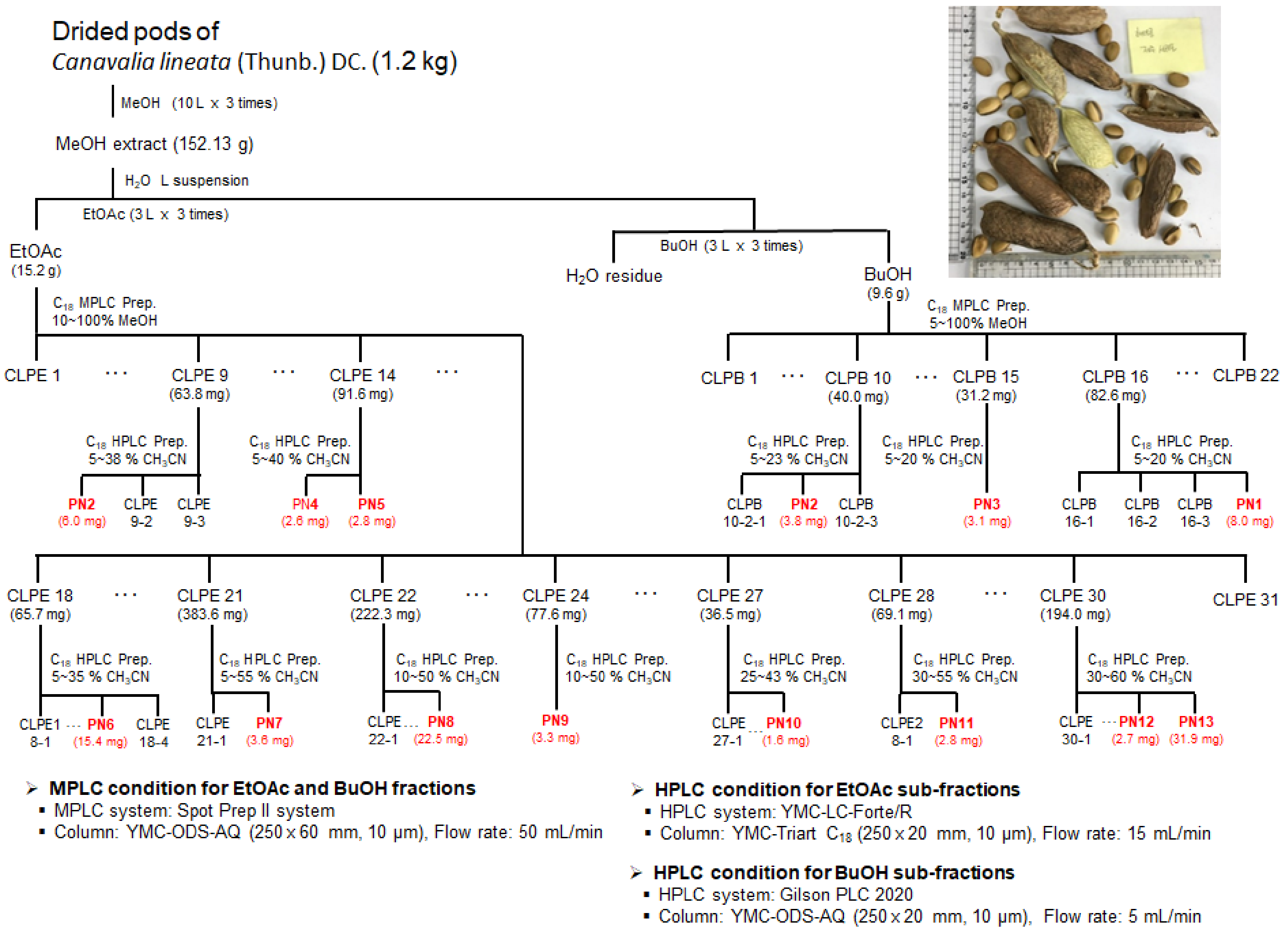

Figure S1. Separation scheme of compounds (**PN1–PN13**) from *Canavalia lineata* pods.

2

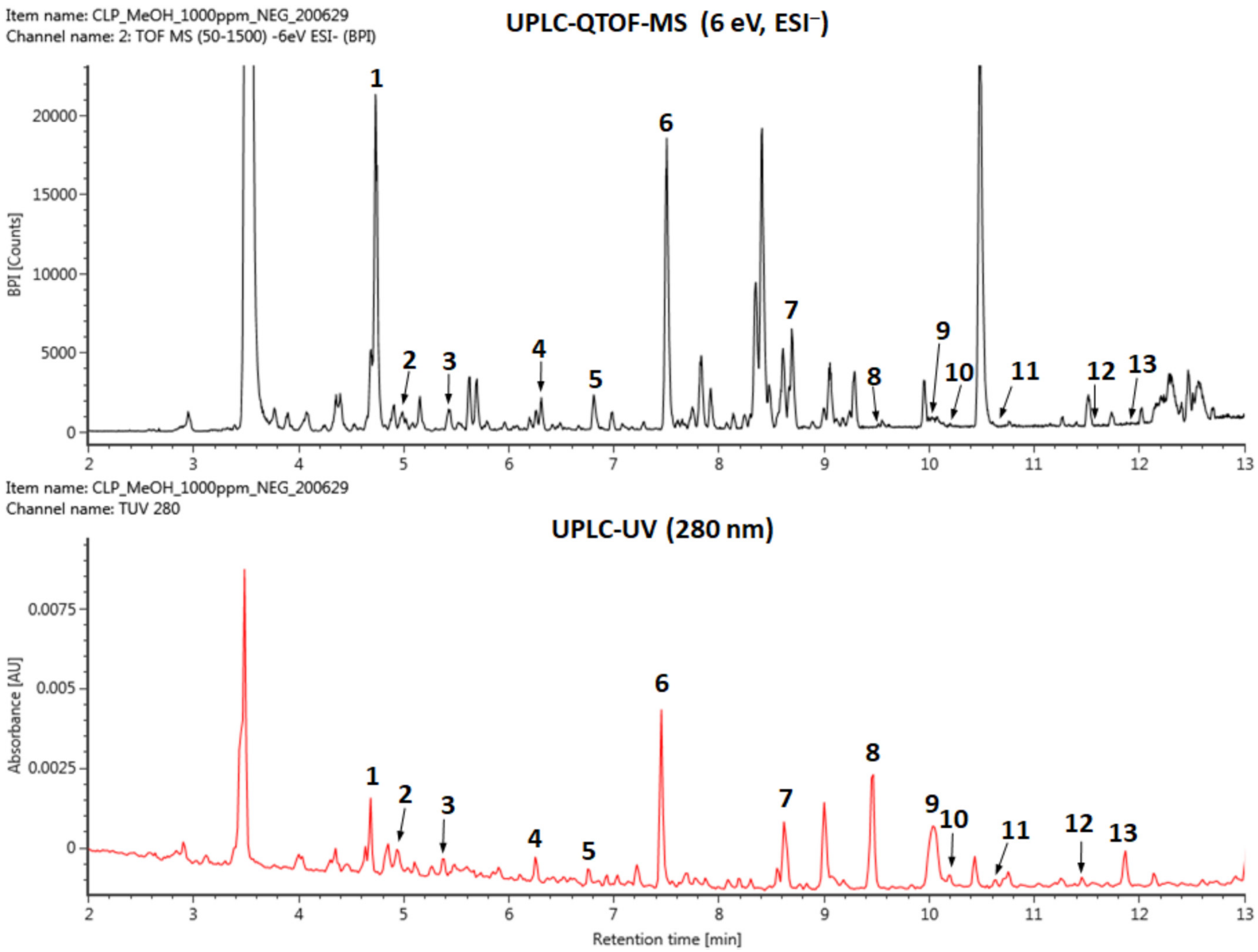

Figure S2. MS and UV chromatogram of *C. lineata* pod extract using UPLC-QTOF-MS.

PN 1. Rutin

Mass	Calc. Mass	mDa	PPM	DBE	Formula	C	H	O
611.1608	611.1612	-0.4	-0.7	12.5	C27 H31 O16	27	31	16
	611.1647	-3.9	-6.4	34.5	C45 H23 O3	45	23	3
	611.1553	5.5	9.0	21.5	C34 H27 O11	34	27	11

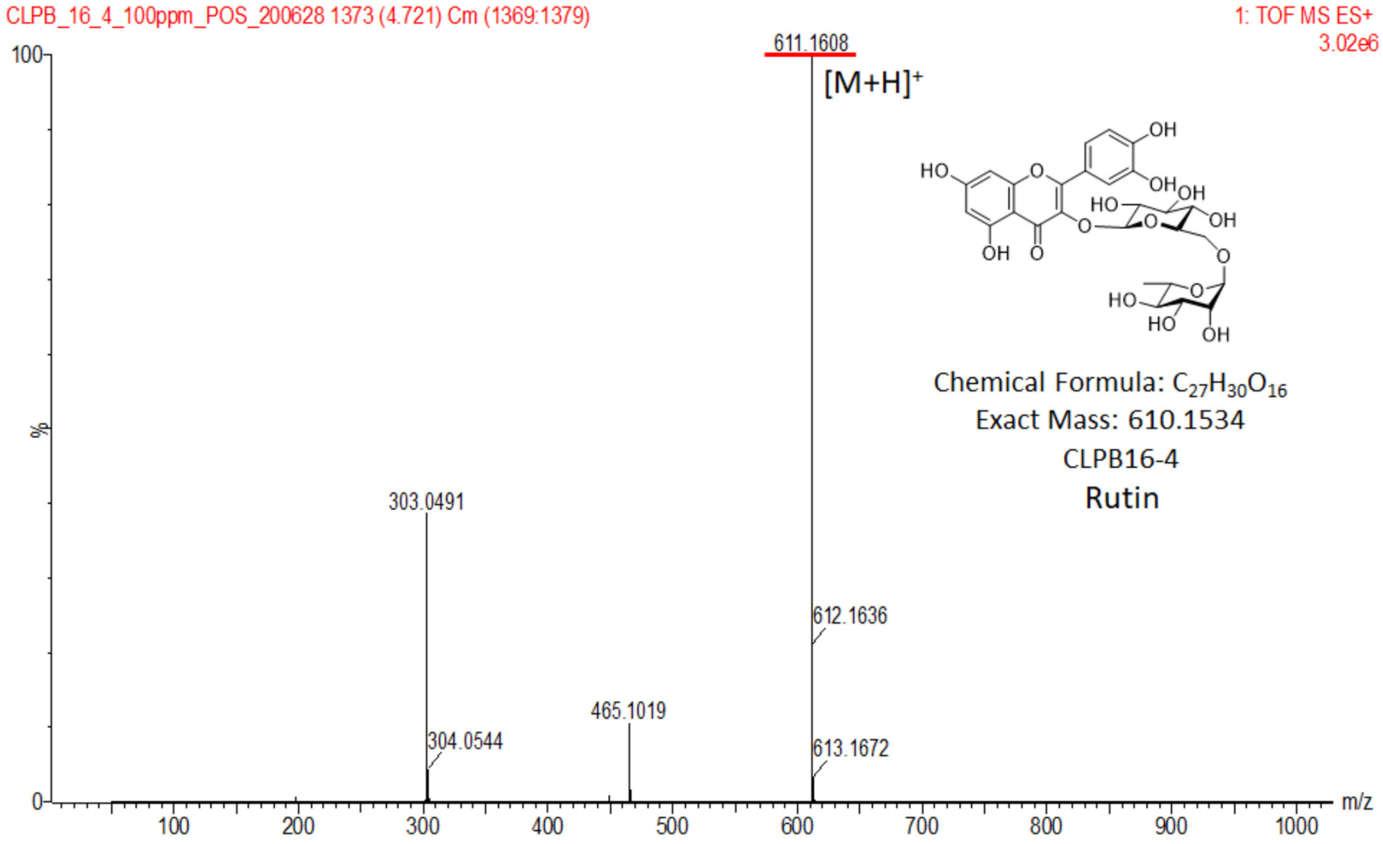

Figure S3. UPLC-QTOF-MS spectrum of rutin (**1**).

PN 2. (2*R*,3*R*)-3-hydroxy 7-*O*-D-glucopyranoside-6-methoxy-flavanone

Mass	Calc. Mass	mDa	PPM	DBE	Formula	C	H	O
493.1286	493.1287	-0.1	-0.2	20.5	C30 H21 O7	30	21	7
	493.1252	3.4	6.9	-1.5	C12 H29 O20	12	29	20
	493.1229	5.7	11.6	29.5	C37 H17 O2	37	17	2
	493.1346	-6.0	-12.2	11.5	C23 H25 O12	23	25	12
	493.1193	9.3	18.9	7.5	C19 H25 O15	19	25	15

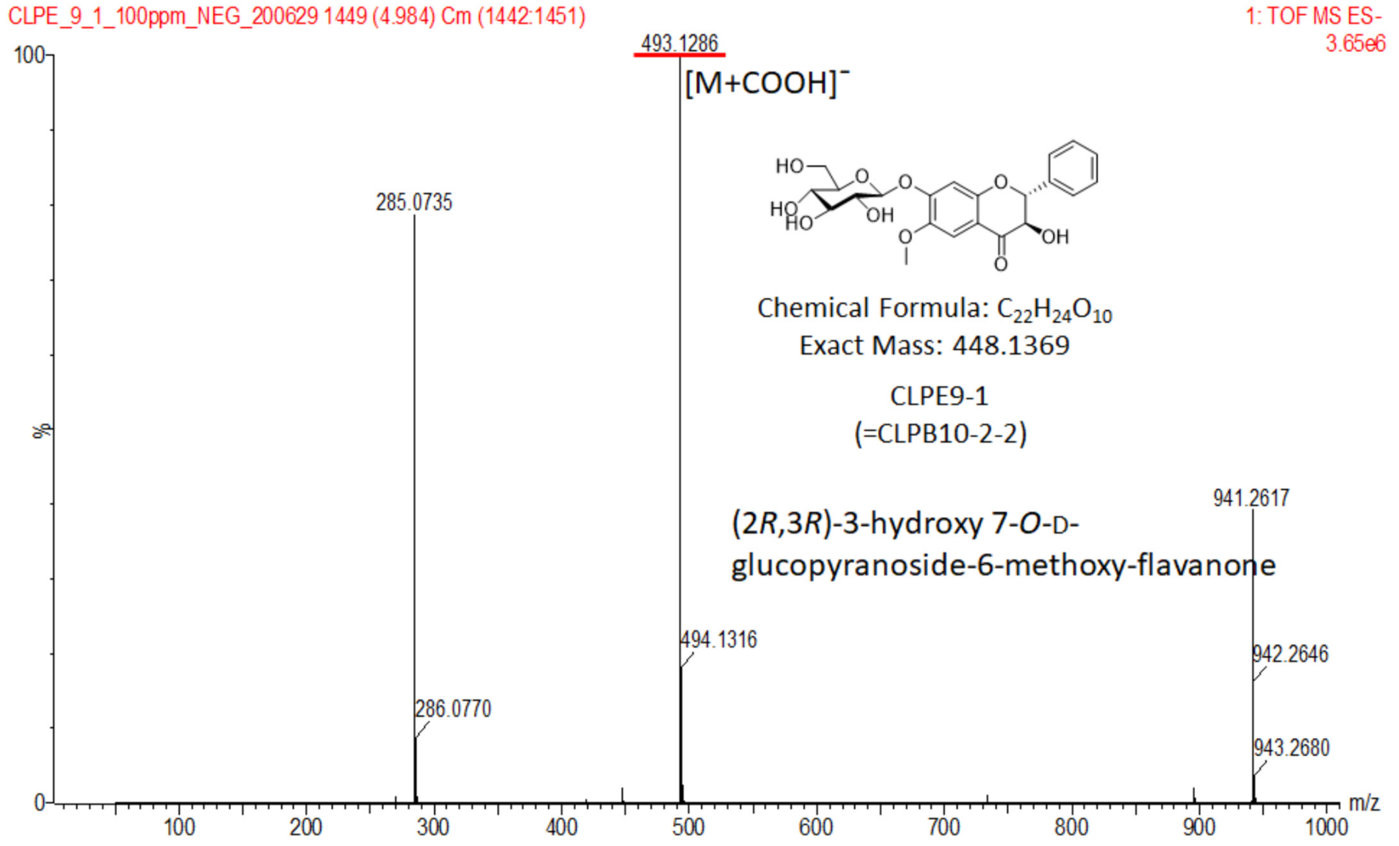

Figure S4. UPLC-QTOF-MS spectrum of (2*R*,3*R*)-3-hydroxy 7-*O*-*β*-D-glucopyranoside-6-methoxy-flavanone (**2**).

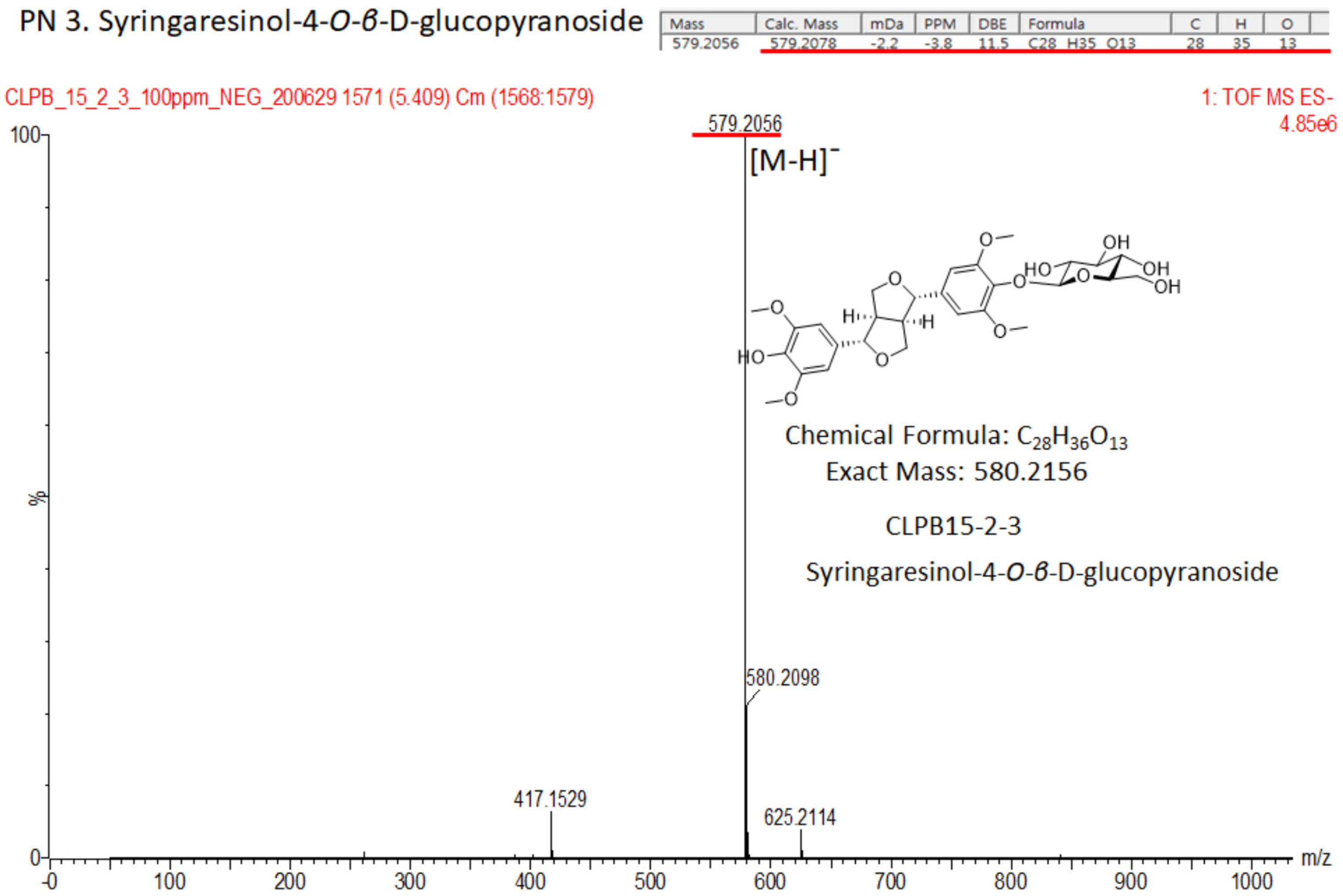

Figure S5. UPLC-QTOF-MS spectrum of (–)-syringaresinol-4-*O*-*β*-D-glucopyranoside (**3**).

6

PN 4. Ononin

Mass	Calc. Mass	mDa	PPM	DBE	Formula	C	H	O
431.1305	431.1283	2.2	5.1	20.5	C29 H19 O4	29	19	4
	431.1342	-3.7	-8.6	11.5	C22 H23 O9	22	23	9
	431.1248	5.7	13.2	-1.5	C11 H27 O17	11	27	17
	431.1401	-9.6	-22.3	2.5	C15 H27 O14	15	27	14
	431.1190	11.5	26.7	7.5	C18 H23 O12	18	23	12

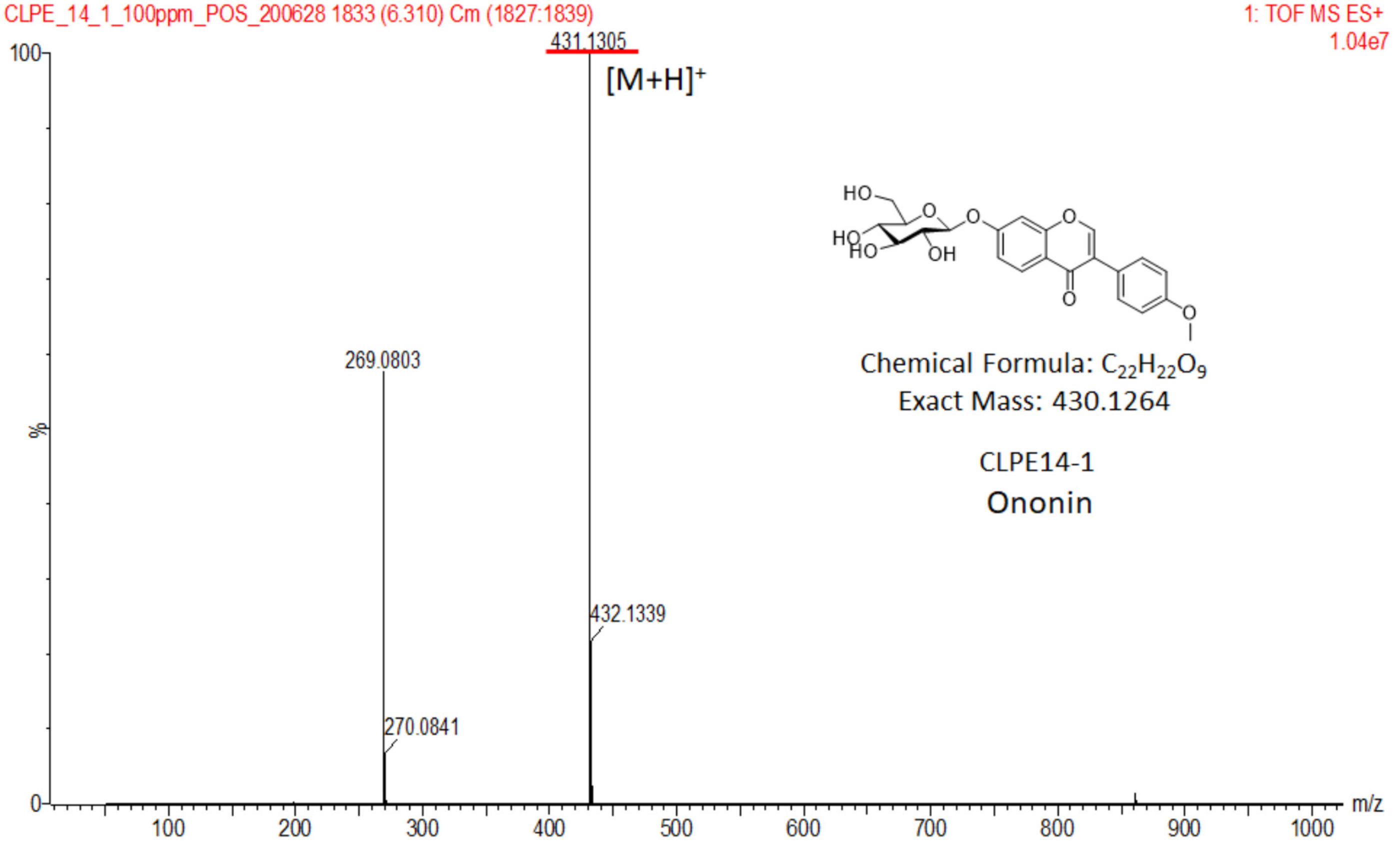

Figure S6. UPLC-QTOF-MS spectrum of ononin (**4**).

7

PN 5. Syringaresinol

Mass	Calc. Mass	mDa	PPM	DBE	Formula	C	H	O
419.1668	419.1647	2.1	5.0	18.5	C29 H23 O3	29	23	3
	419.1706	-3.8	-9.1	9.5	C22 H27 O8	22	27	8
	419.1765	-9.7	-23.1	0.5	C15 H31 O13	15	31	13
	419.1553	11.5	27.4	5.5	C18 H27 O11	18	27	11

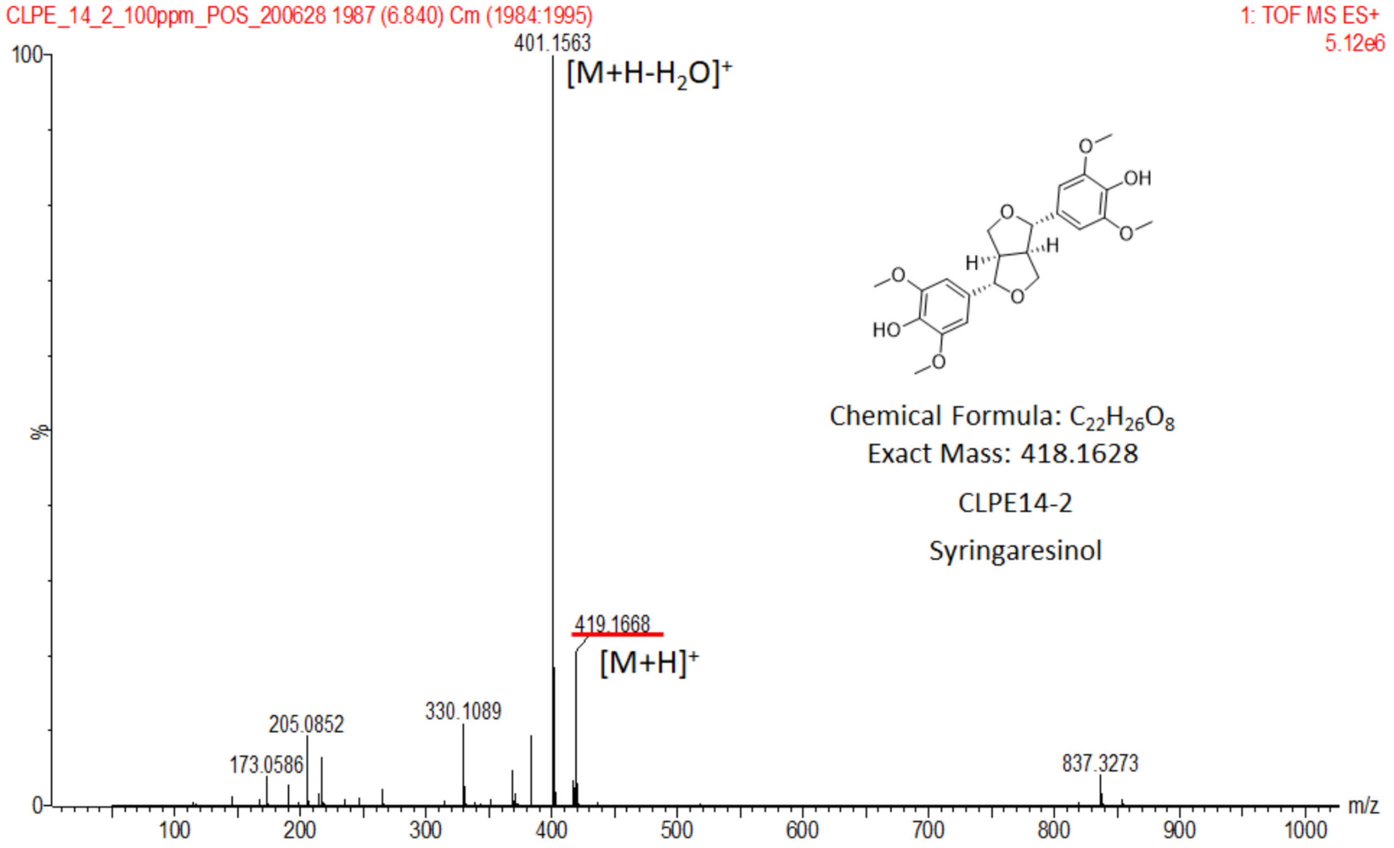

Figure S7. UPLC-QTOF-MS spectrum of syringaresinol (**5**).

8

PN 6. (2*R*,3*R*)-3,7'-Dihydroxy-6-methoxy-flavanone

Mass	Calc. Mass	mDa	PPM	DBE	Formula	C	H	O
287.0902	287.0919	-1.7	-5.9	9.5	C16 H15 O5	16	15	5
	287.0861	4.1	14.3	18.5	C23 H11	23	11	
	287.0978	-7.6	-26.5	0.5	C9 H19 O10	9	19	10

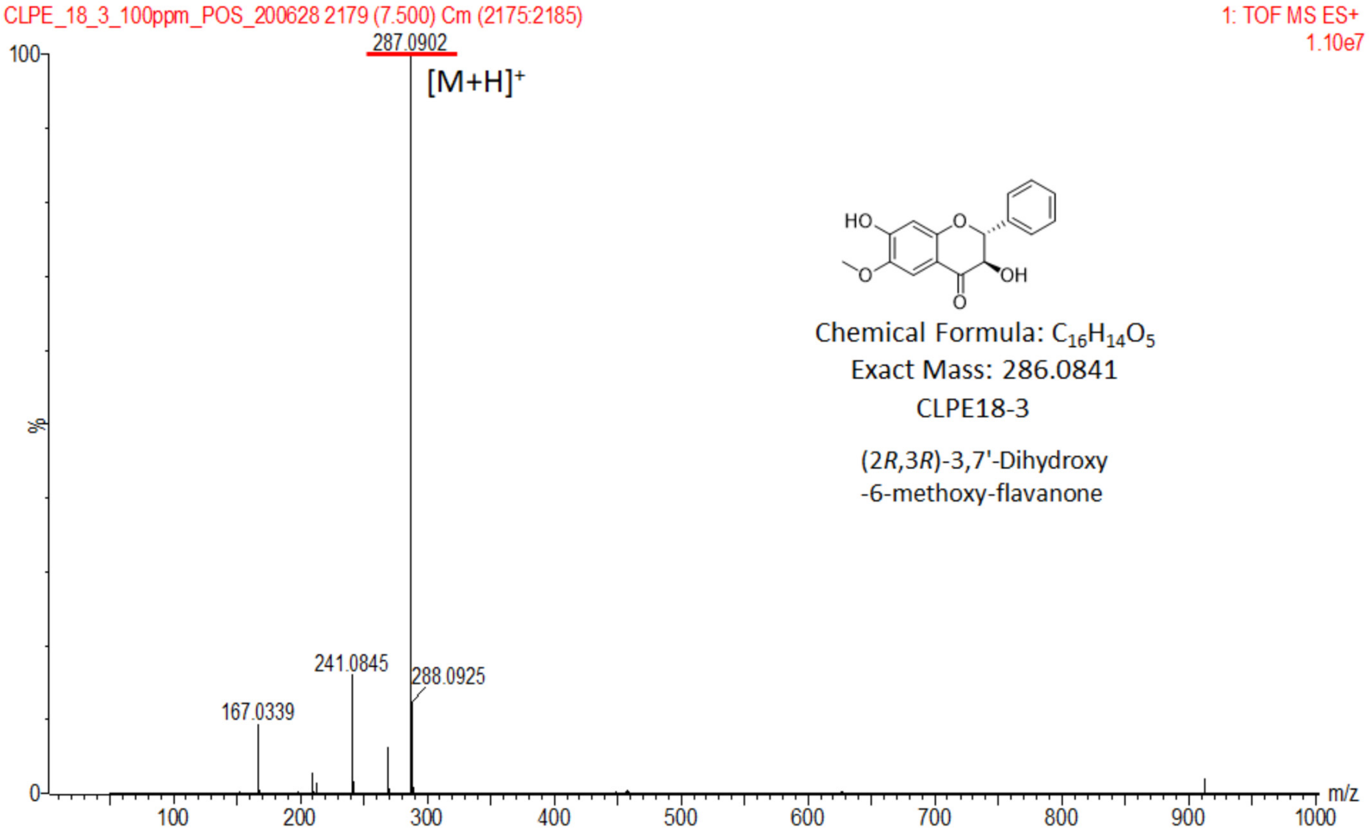

Figure S8. UPLC-QTOF-MS spectrum of (2*R*,3*R*)-3,7-dihydroxy-6-methoxyflavanone (**6**).

PN 7. Cajanin

Mass	Calc. Mass	mDa	PPM	DBE	Formula	C	H	O
301.0555	301.0560	-0.5	-1.7	6.5	C12 H13 O9	12	13	9
	301.0501	5.4	17.9	15.5	C19 H9 O4	19	9	4
	301.0653	-9.8	-32.6	19.5	C23 H9 O	23	9	1
	301.0407	14.8	49.2	2.5	C8 H13 O12	8	13	12
	301.0712	-15.7	-52.1	10.5	C16 H13 O6	16	13	6

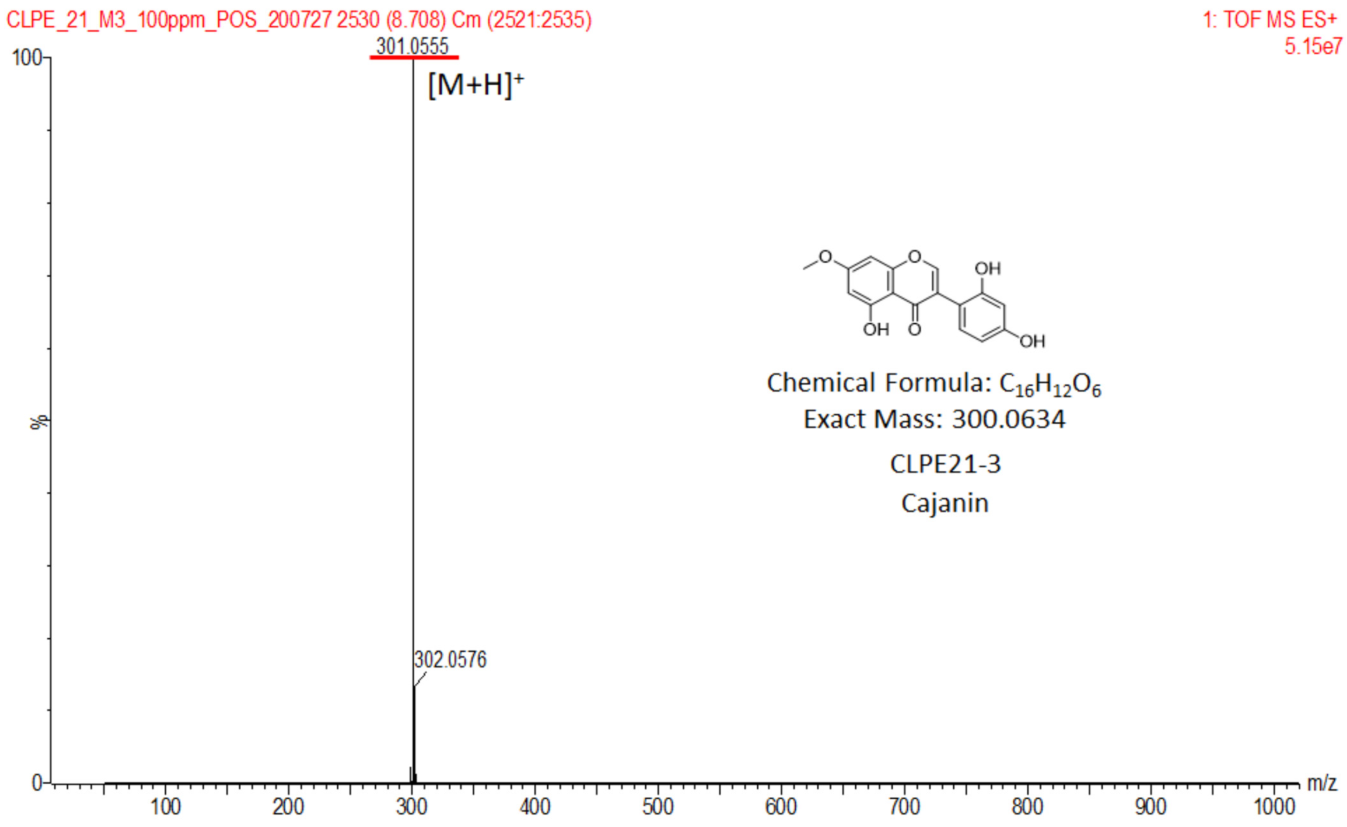

Figure S9. UPLC-QTOF-MS spectrum of cajanin (**7**).

PN 8. Medicarpin

Mass	Calc. Mass	mDa	PPM	DBE	Formula	C	H	O
271.0940	271.0970	-3.0	-11.1	9.5	C16 H15 O4	16	15	4
	271.1029	-8.9	-32.8	0.5	C9 H19 O9	9	19	9
	271.0818	12.2	45.0	5.5	C12 H15 O7	12	15	7

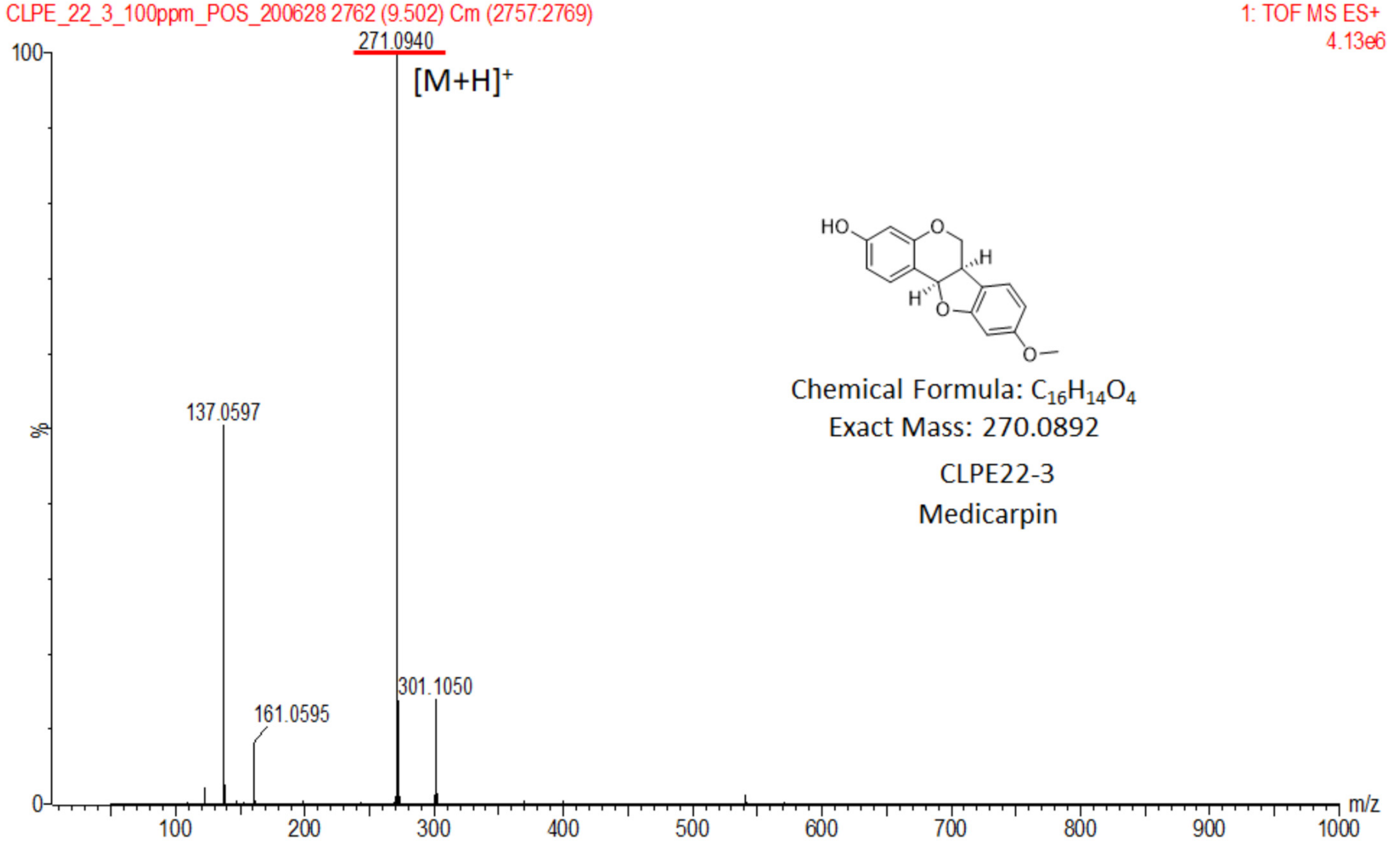

Figure S10. UPLC-QTOF-MS spectrum of medicarpin (**8**).

11

PN 9. Prunetin

Mass	Calc. Mass	mDa	PPM	DBE	Formula	C	H	O
285.0736	285.0763	-2.7	-9.5	10.5	C16 H13 O5	16	13	5
	285.0704	3.2	11.2	19.5	C23 H9	23	9	

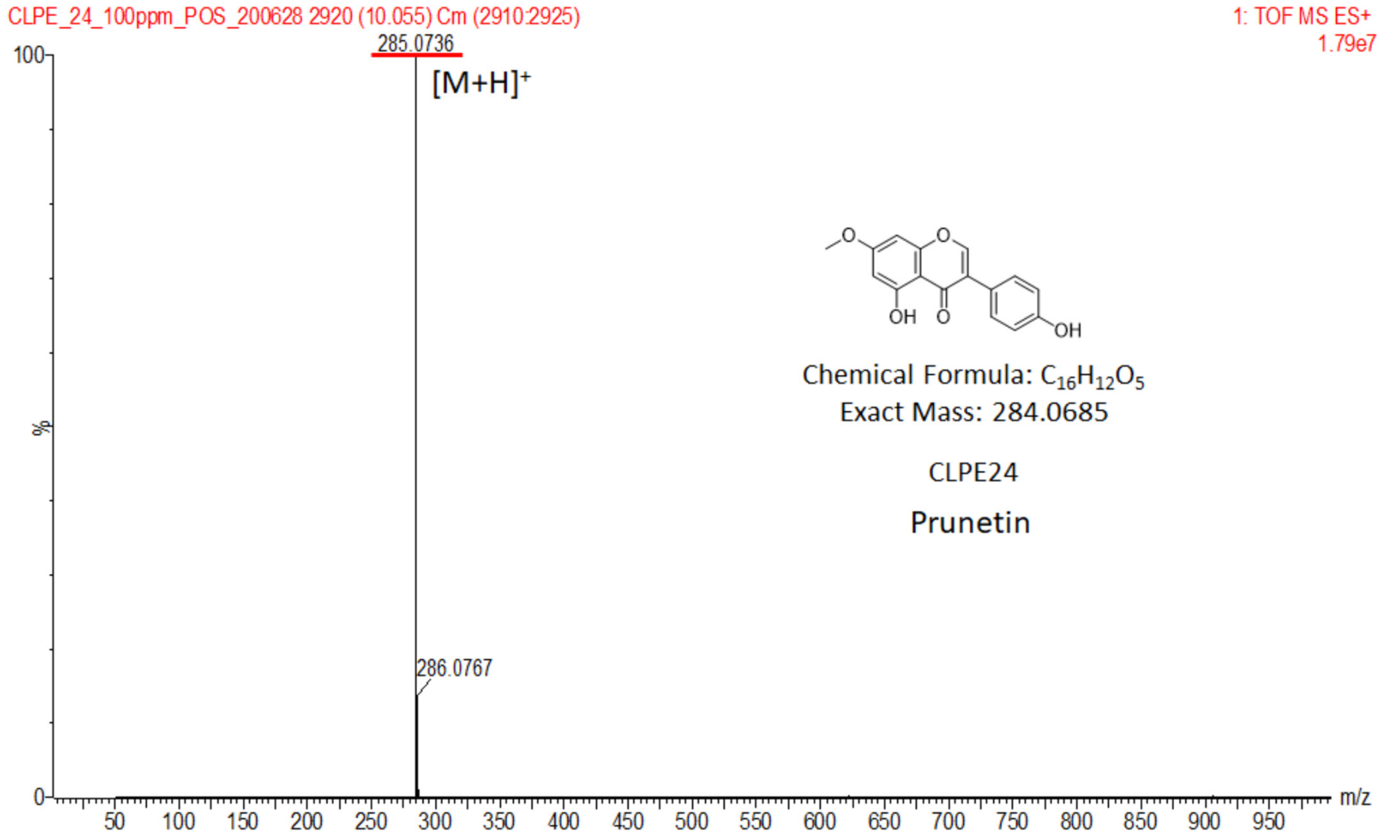

Figure S11. UPLC-QTOF-MS spectrum of prunetin (**9**).

12

PN 10. 7,4'-Dimethyl-3'-hydroxygenistein

Mass	Calc. Mass	mDa	PPM	DBE	Formula	C	H	O
315.0837	315.0810	2.7	8.6	19.5	C24 H11 O	24	11	1
	315.0869	-3.2	-10.2	10.5	C17 H15 O6	17	15	6
	315.0927	-9.0	-28.6	1.5	C10 H19 O11	10	19	11

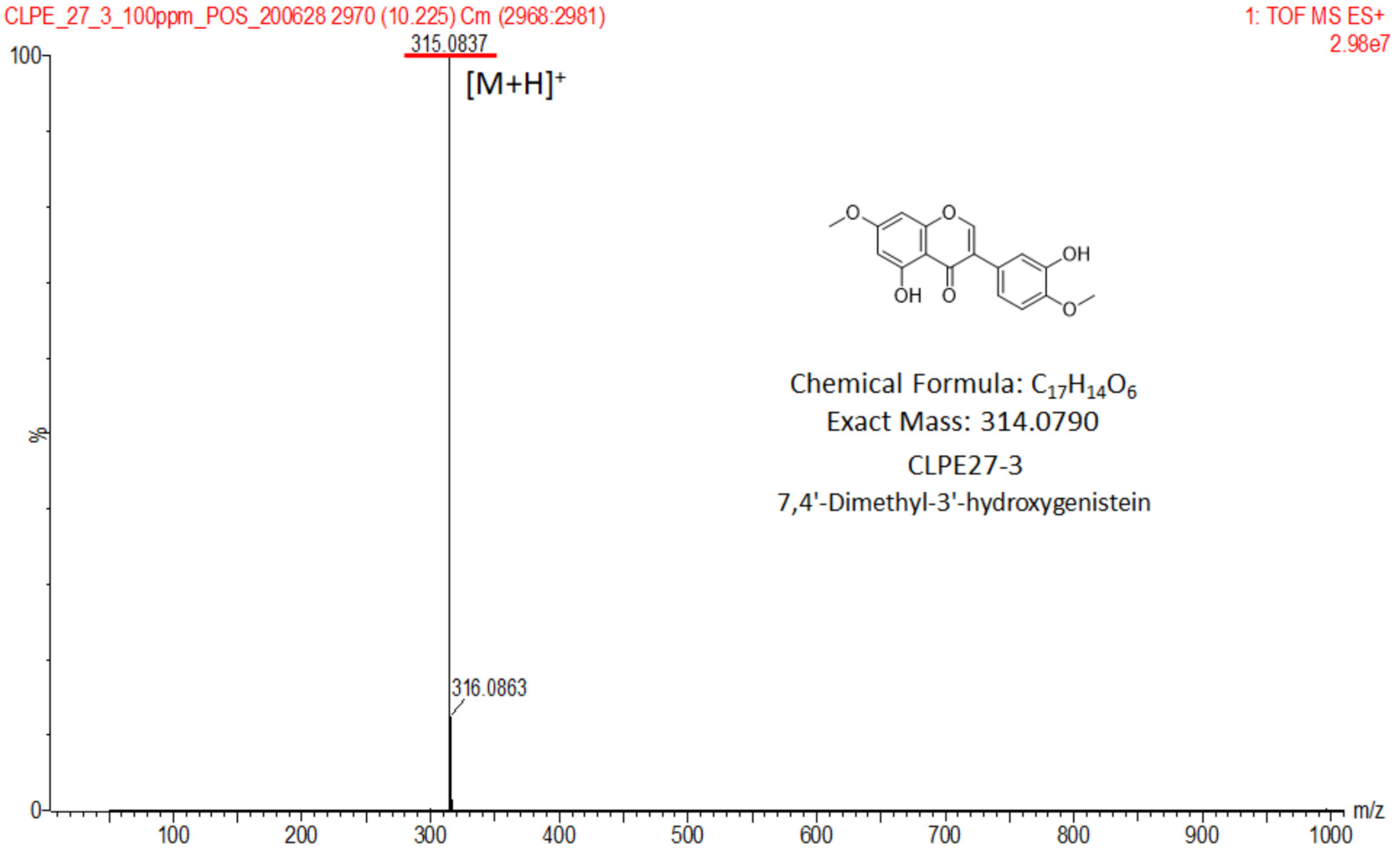

Figure S12. UPLC-QTOF-MS spectrum of 7,4'-dimethyl-3'-hydroxygenistein (**10**).

13

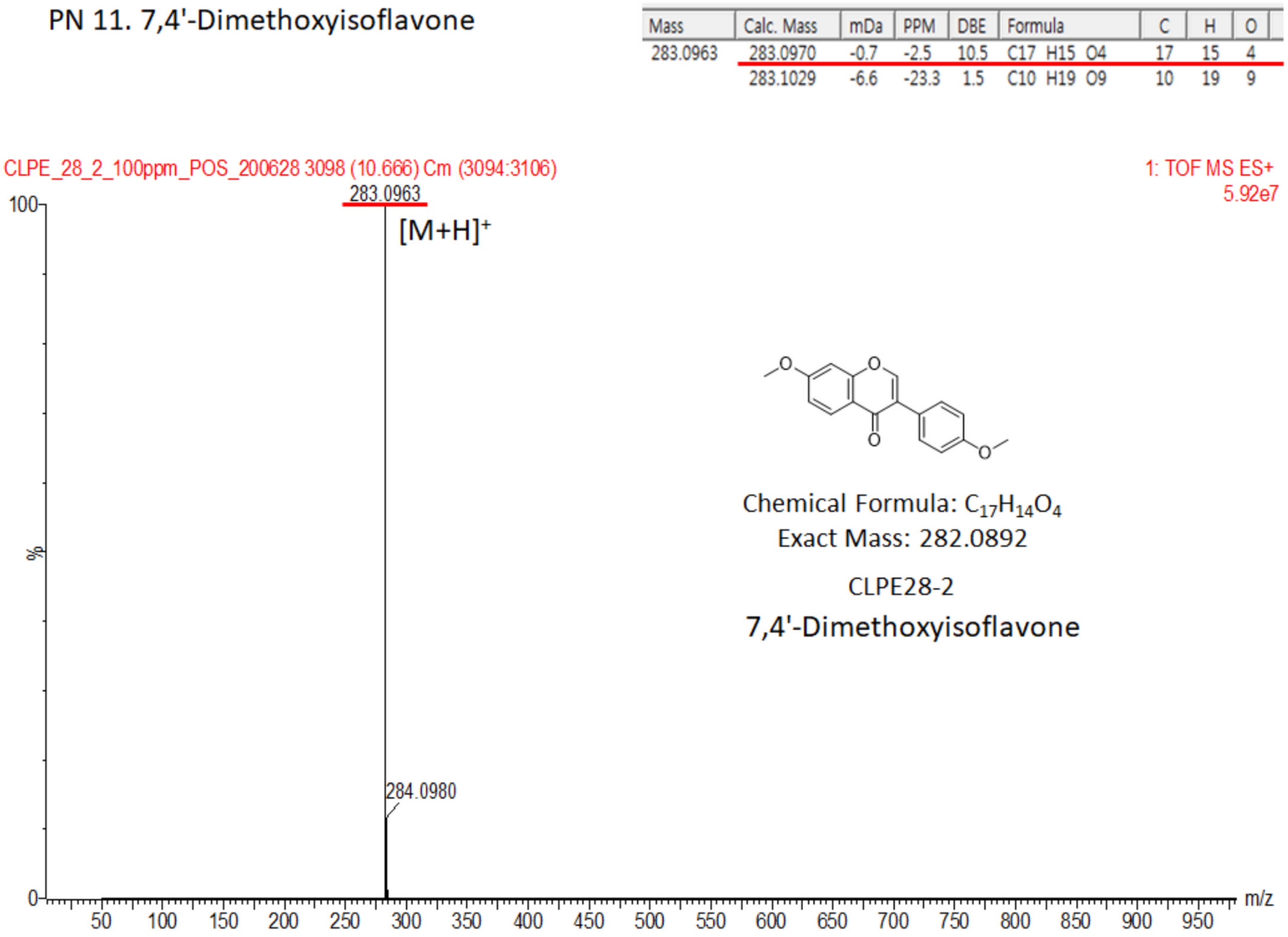

Figure S13. UPLC-QTOF-MS spectrum of 7,4'-dimethoxyisoflavone (**11**).

14

PN 12. Pterocarpin

Mass	Calc. Mass	mDa	PPM	DBE	Formula	C	H	O
299.0903	299.0919	-1.6	-5.3	10.5	C17 H15 O5	17	15	5
	299.0861	4.2	14.0	19.5	C24 H11	24	11	

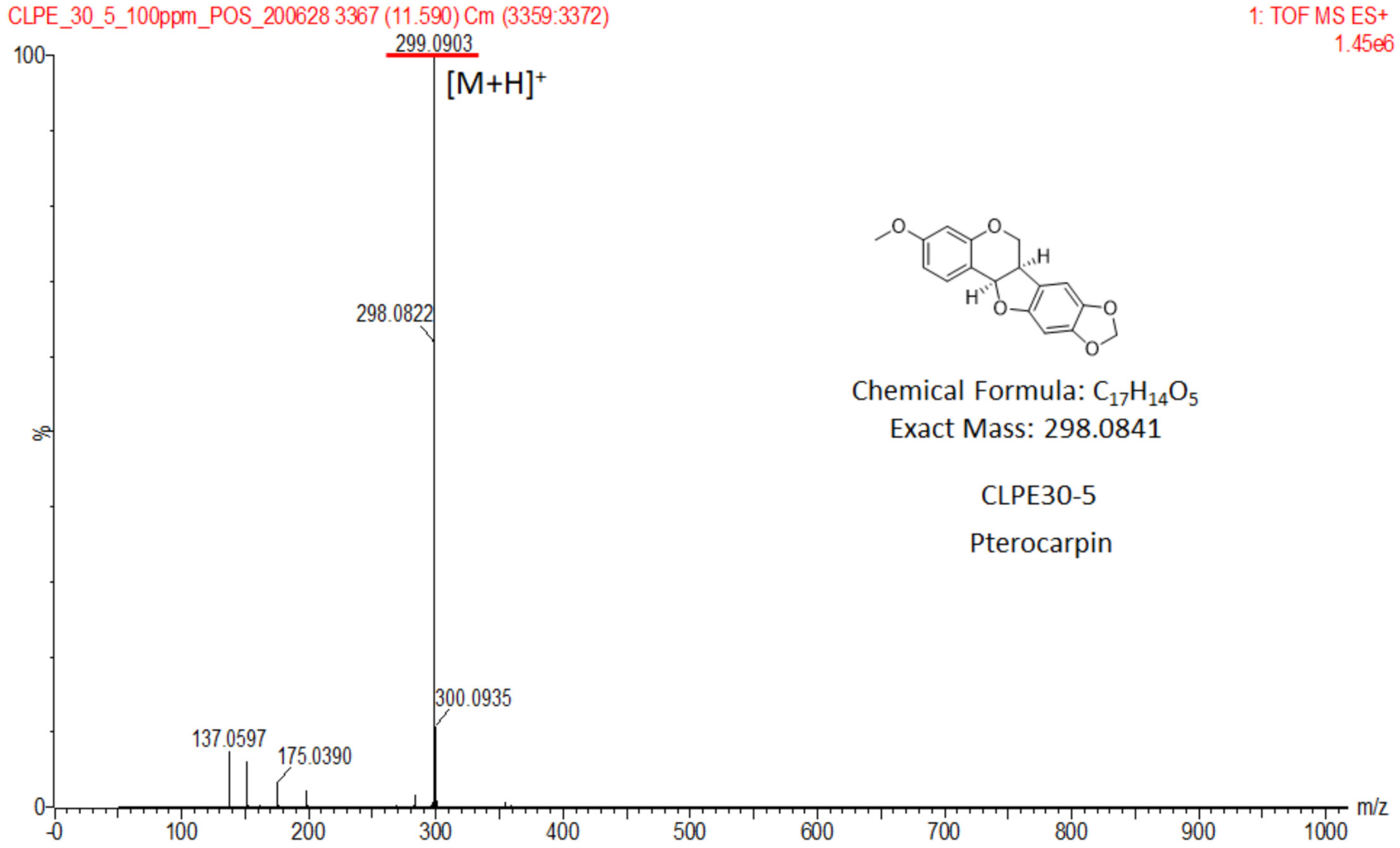

Figure S14. UPLC-QTOF-MS spectrum of pterocarpin (**12**).

PN 13. Homopterocarpin

Mass	Calc. Mass	mDa	PPM	DBE	Formula	C	H	O
285.1123	285.1127	-0.4	-1.4	9.5	C17 H17 O4	17	17	4
	285.1186	-6.3	-22.1	0.5	C10 H21 O9	10	21	9
	285.0974	14.9	52.3	5.5	C13 H17 O7	13	17	7
	285.1279	-15.6	-54.7	13.5	C21 H17 O	21	17	1

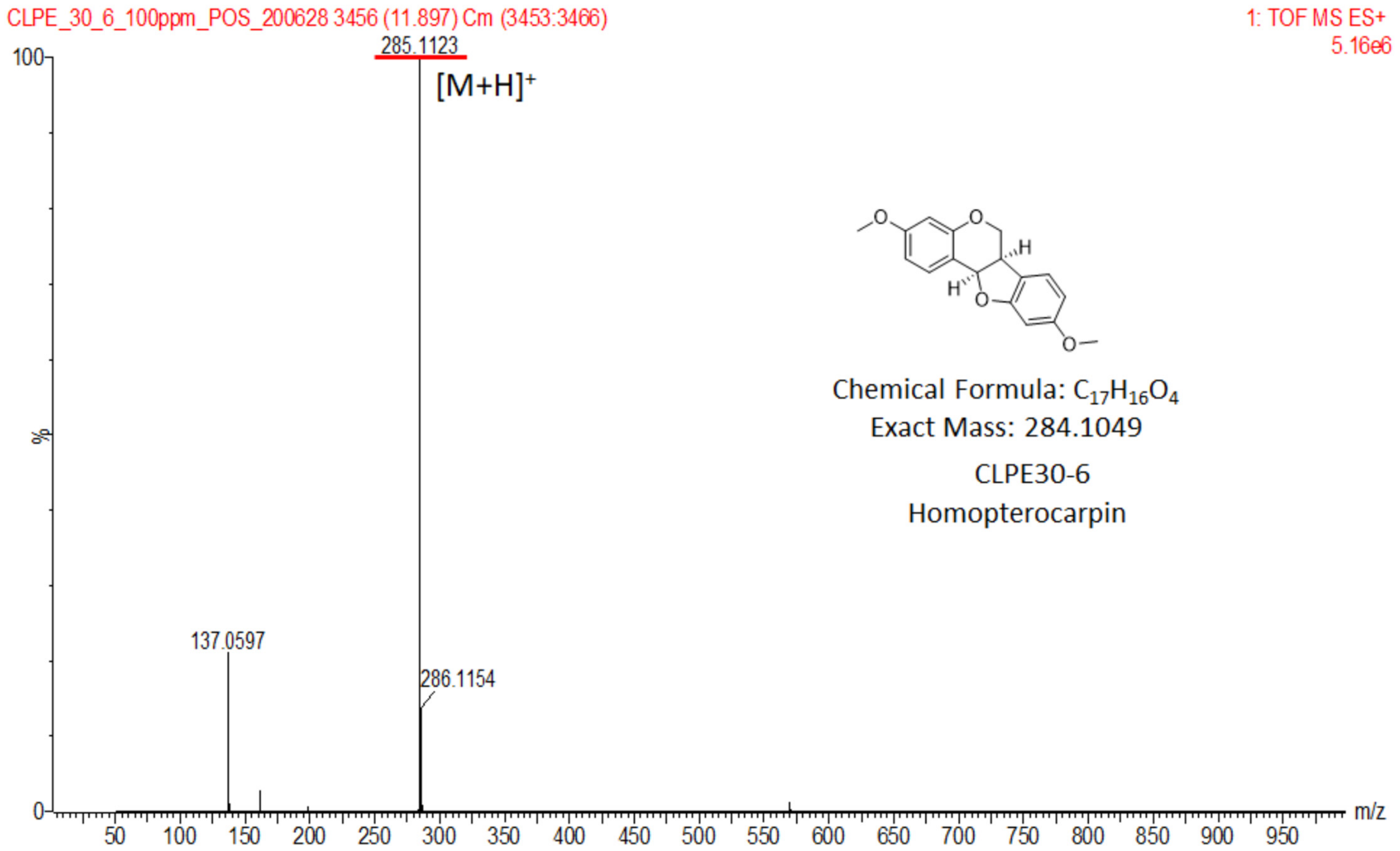

Figure S15. UPLC-QTOF-MS spectrum of homopterocarpin (**13**).

16

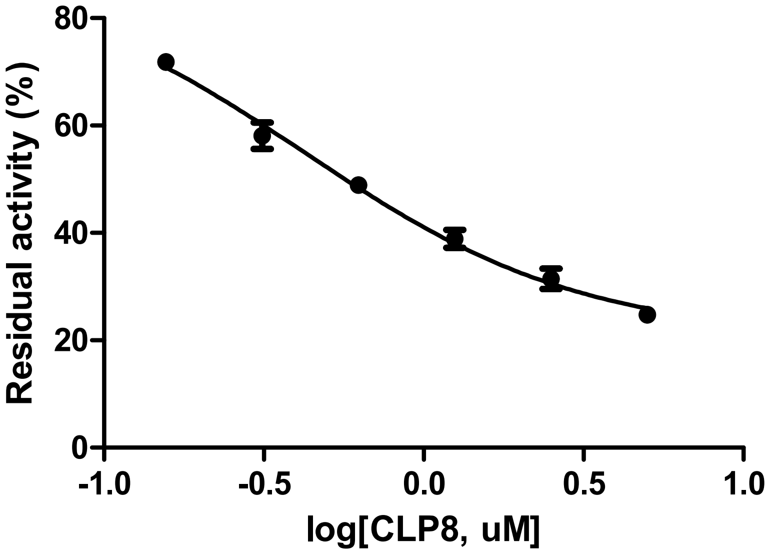

Figure S16. IC_{50} curve of medicarpin (**8**) for hMAO-B.

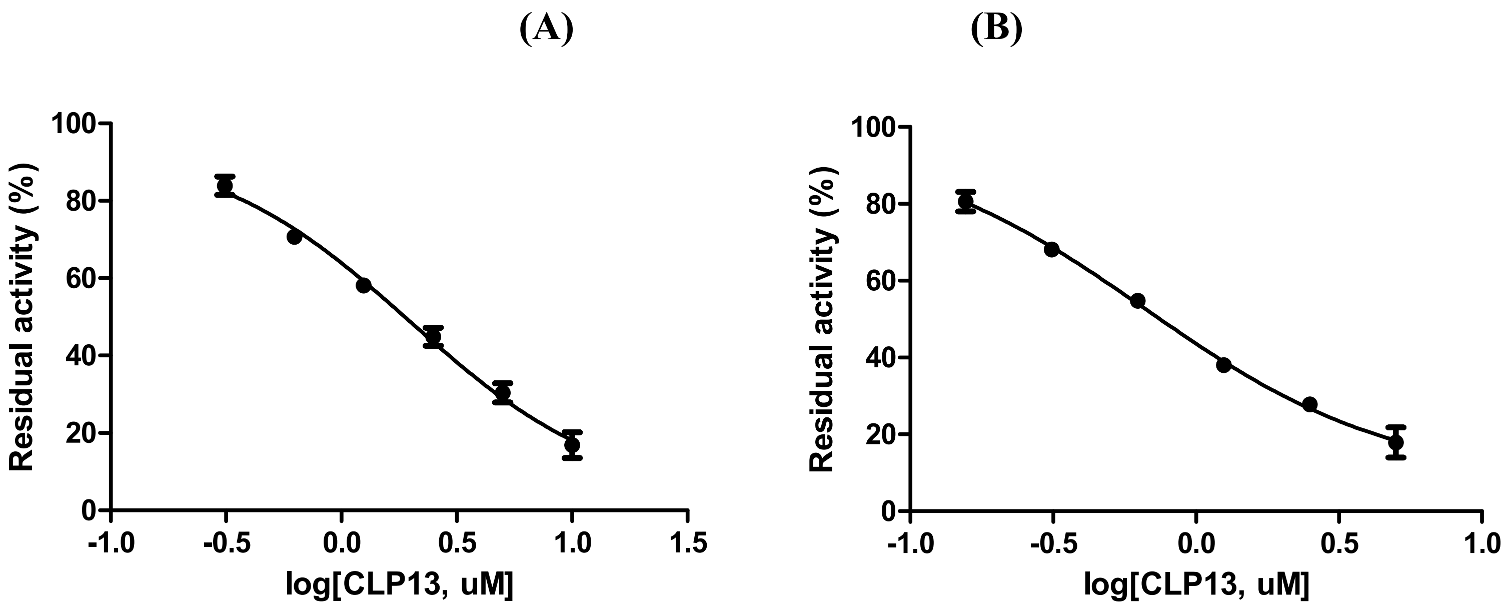

Figure S17. IC_{50} curves of homopterocarpin (**13**) for hMAO-A (**A**) and hMAO-B (**B**).
